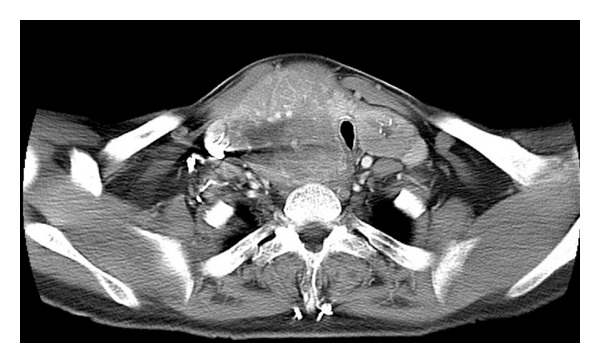# Localized Advanced Hürthle Cell Carcinoma with Symptomatic Intrathoracic Goiter

**DOI:** 10.1155/2011/623130

**Published:** 2011-09-29

**Authors:** Kabir Ahmed, Darren Swartz, Deepu Daniel, Craig Crespi, Andrew Rosenthal, Joseph DeCostanza

**Affiliations:** Division of Trauma Services, Memorial Regional Hospital, 3501 Johnson Street, Hollywood, FL 33021, USA

## Abstract

Intrathoracic goiters are divided into two categories: primary and secondary. Intrathoracic goiters (IG) can cause upper airway obstruction. The presence of obstructive symptoms secondary to increased thyroid growth and tracheal compression is major indication for surgery; however, goiters do not always require immediate surgical attention. In addition, although some diagnostic tests indicate upper airway obstruction, many patients remain asymptomatic. Surgeries to remove IG are performed routinely however, they are not without risk. In some cases, intrathoracic goiters present as thyroid cancers. Very rare cancers such as Hürthle cell carcinoma (HCC) can create a challenge for the surgeon when surgical intervention is vital.

## 1. Introduction

Intrathoracic goiters can be divided into primary or secondary intrathoracic goiters depending on the origin of their blood supply. Primary intrathoracic goiters are exceedingly rare, and their blood supply is produced from intrathoracic origin of vascular pedicle [1]. Secondary intrathoracic goiters are common, and their blood supply originates from cervical vascular pedicle.

Major indications for surgery include the presence of obstructive symptoms (i.e., dysphagia, exertional dyspnea, and cough) secondary to increased thyroid growth and tracheal compression. Enlargement of intrathoracic goiters beyond the brachiocephalic vein heralds the consideration of surgery as a treatment option. Other indications for surgery include continued growth of goiter as seen in computed axial tomography scans (CT). There are specific reasons for surgery in asymptomatic individuals. These reasons include increased difficulty in removing intrathoracic goiters once patients become symptomatic, ineffectiveness of hormone suppressive therapy, increased risk of surgical complications with age, possibility of cancer within the intrathoracic goiter which may prove difficult to biopsy or palpate, and the risk of hemorrhage into goiter which may lead to acute airway obstruction. Approximately 42% of patients who show evidence of upper airway obstruction on flow volume loops are asymptomatic, making surgery a primary mode of treatment in such individuals.

## 2. Case Presentation

**Figure 1:**
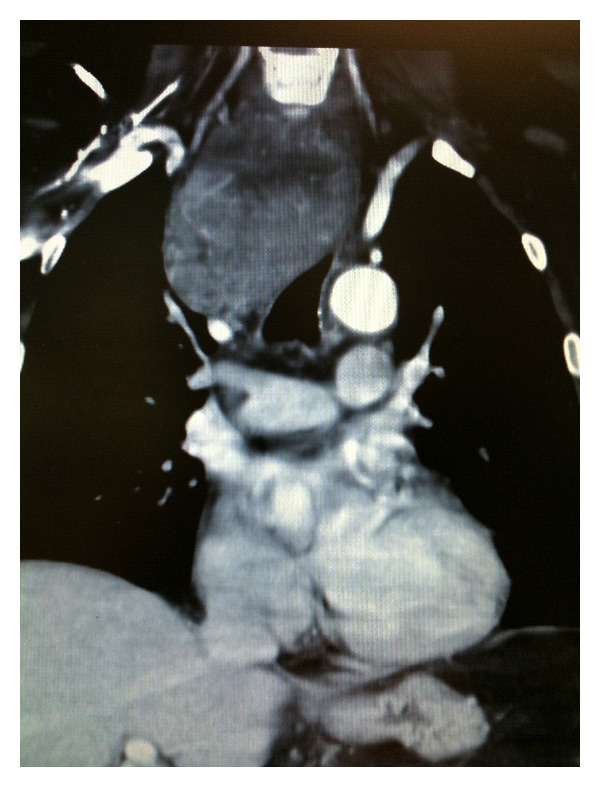


A 50-year-old male presented to the emergency room complaining of dyspnea. The patient described his dyspnea as a smothering sensation in his neck and tightness in his chest. The patient was expectorating purulent green sputum and experiencing chills. For several months prior to admission, the patient stated that he had developed worsening orthopnea, dyspnea, and enlargement of a goiter to the extent that he could not button his shirt collar. The patient stated that he noticed a right neck mass in midline of his neck which started approximately three years ago. Since then, the mass had slowly grown to the lateral portion of the right neck below the right ear and to a lesser degree on the left. The patient did not previously seek medical attention because of lack of health insurance. All laboratory tests, including thyroid function tests, complete blood count, and coagulation studies, were within normal limits. Upon admission, the patient underwent computed tomography scan (CT) demonstrating a large thyroid mass measuring 8 × 6 × 9 × 17 cm, extending to the level of the aortic arch and carina with significant retroesophageal lobules, in addition to compression and displacement of the trachea and esophagus to the left and compression of the jugular veins (Figure 1). Near-total cervical and mediastinal resection was performed leaving a remnant less than 1 gram in the right superior pole. The final histology report of the specimens demonstrated HCC.

**Figure 2:**
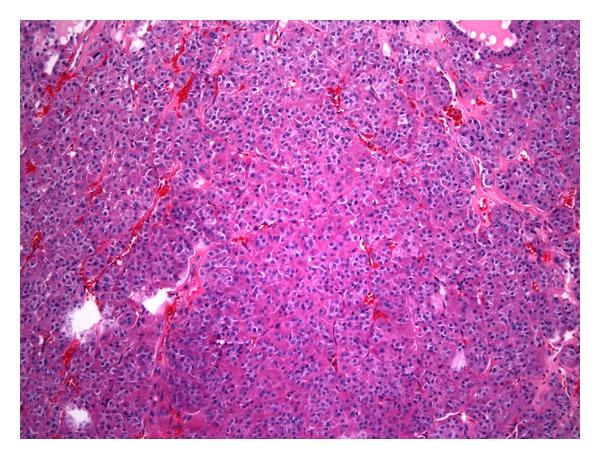


During a follow-up appointment, a posterior triangular mass was discovered which seemed distinct from the previous goiter. Originally thought to be a lymph node, a fine-needle biopsy was performed which was consistent with HCC (Figure 2). The patient was brought back to the operating room for the excision of this mobile but firm 2.5 × 2.5 cm enlarged mass resulting in an additional focus of HCC.

## 3. Discussion

Primarily composed of Hürthle cells and considered to be a variant of follicular carcinoma (oxyphilic type), HCC accounts for a small percentage of all differentiated thyroid cancers. HCCs are often multifocal, bilateral, euthyroid, and larger than benign adenomas (3.1 cm versus 1.9 cm) [2]. In addition, these encapsulated neoplasms are typically hypercellular with minimal to no lymphocytes and colloid. They are also known to be more aggressive than follicular cells and differ from the benign type by presence of vascular and capsular invasion, and extrathyroidal spread. Unfortunately, recurring HCCs are aggressive and often incurable [3].

The preoperative workup includes a history and physical examination, laboratory testing, radiology studies, flow volume loop test, and fine-needle aspiration. A thorough physical exam is indicated in any preoperative workup. Specifically, the presence of IG can be indicated by the inability of the examiner to palpate the lower end of the thyroid gland. The examiner may also note tracheal deviation and dilated neck veins. Physicians may utilize the Pemberton's maneuver in further evaluation of intrathoracic goiter.

Pemberton's maneuver requires the examiner to have the patient raised his or her hands above their head for 60 seconds. The maneuver is used to demonstrate obstructive symptoms by forcing the thyroid gland into thoracic inlet. A positive test is indicated by neck vein distension, facial flushing, cyanosis, dysphagia, and worsening dyspnea. A thyroid stimulating hormone (TSH) level should be obtained in all patients with goiter. Radiologic workup includes chest X-rays, ultrasound, CT, and occasionally magnetic resonance imaging (MRI). Chest X-rays can show the presence of intrathoracic goiter, and consequent compression and deviation of the trachea. It is optimal to compare previous chest X-rays in order to look for patterns of increased growth of intrathoracic goiters. CT and MRI allow for further assessment of intrathoracic goiter, its extension into substernal region, and compression of adjacent structures (Figure 3).

The entire vertical length of the goiter must be imaged when obtaining a CT or MRI. This is done with the patient's neck in neutral or slightly flexed positions. The flow volume loop test is uncommonly performed to evaluate intrathoracic goiter. This test can be utilized to indicate upper airway obstruction. A blunted flow volume loop is produced by intrathoracic goiters, indicating an equal limitation of flow in both inspiration and expiration. Fine-needle aspirations are indicated in the presence of discrete nodule, rapid growth, pain/tenderness, and hardness in one region of the goiter.

The standard cervical incision is used in the majority of intrathoracic goiters. A partial or complete sternotomy may be necessary in individuals with history of prior cervical thyroidectomy, large intrathoracic goiter, invasive carcinoma, recurrent goiter, and ectopic goiter. An isthmusectomy can be performed in patients with chronic autoimmune thyroiditis who present with concentric tracheal compression. Isthmusectomy allows for alleviation of obstructive symptoms. Separating these inflamed glands from surrounding nerves and parathyroid glands is difficult; therefore, isthmusectomy can avoid unwanted complications from further surgery. 

Major surgical complications include injury to recurrent laryngeal nerves, trachea, and parathyroid glands. Recurrent laryngeal nerve damage is seen to occur in two to nine percent of patients undergoing thyroid surgery [4]. Hypocalcemia is the most common complication of near total thyroidectomy secondary to removal of parathyroid glands. Hypocalcemia is more commonly seen in cases of extensive goiters such as intrathoracic goiters. Tracheomalacia is another major complication that can occur in thyroidectomy. Goiters can cause pressure-induced destruction of tracheal rings. This leads to postoperative collapse of airways. These patients cannot be extubated immediately after surgery but are generally extubated by day ten [4].

## Figures and Tables

**Figure 3:**